# In Vitro Models of Biological Barriers for Nanomedical Research

**DOI:** 10.3390/ijms23168910

**Published:** 2022-08-10

**Authors:** Flavia Carton, Manuela Malatesta

**Affiliations:** 1 Department of Health Sciences, University of Piemonte Orientale “A. Avogadro”, 28100 Novara, Italy; 2 Department of Neurosciences, Biomedicine and Movement Sciences, University of Verona, 37134 Verona, Italy

**Keywords:** nanoparticles, cell culture, cell monolayer, spheroid, bioreactor, microfluidics

## Abstract

Nanoconstructs developed for biomedical purposes must overcome diverse biological barriers before reaching the target where playing their therapeutic or diagnostic function. In vivo models are very complex and unsuitable to distinguish the roles plaid by the multiple biological barriers on nanoparticle biodistribution and effect; in addition, they are costly, time-consuming and subject to strict ethical regulation. For these reasons, simplified in vitro models are preferred, at least for the earlier phases of the nanoconstruct development. Many in vitro models have therefore been set up. Each model has its own pros and cons: conventional 2D cell cultures are simple and cost-effective, but the information remains limited to single cells; cell monolayers allow the formation of cell–cell junctions and the assessment of nanoparticle translocation across structured barriers but they lack three-dimensionality; 3D cell culture systems are more appropriate to test in vitro nanoparticle biodistribution but they are static; finally, bioreactors and microfluidic devices can mimicking the physiological flow occurring in vivo thus providing in vitro biological barrier models suitable to reliably assess nanoparticles relocation. In this evolving context, the present review provides an overview of the most representative and performing in vitro models of biological barriers set up for nanomedical research.

## 1. Introduction

Nanomedicine is a rapidly progressing field of medical research focusing on the development of nanoconstructs designed for diagnostic, therapeutic, and prophylactic purposes [1]. In view of their intended applications, nanomedical tools need to be thoroughly investigated for their biocompatibility, biodistribution, and effects; therefore, studies on the structural and functional relationships of nanoconstructs with the biological environment—i.e., organs, tissues, and cells—are mandatory. However, living organisms have developed manifold defense mechanisms against exogenous agents, so that a nanoconstruct must overcome diverse biological barriers before reaching the target. The types of biological barriers to be crossed also depend on the administration route, as the nanoconstructs may be delivered intravenously, parenterally, orally, topically, or via inhalation or tracheal instillation [2,3,4,5,6,7,8,9].

Studies on laboratory animals are useful for the preclinical evaluation of nanoparticle (NP) efficacy, but the in vivo models are very complex, and it is often difficult to distinguish the roles plaid by the multiple biological barriers on the NP biodistribution, effect, and clearance. Moreover, animal experiments are costly, time-consuming, and subject to strict ethical regulation [10]. Ex vivo models based on tissue/organ explants from animals raise the same problems as in vivo studies, while explants from humans not only raise ethical issues but, in some cases (e.g., brain samples), are also hardly available.

For all these reasons, simplified in vitro models are preferred at least for the earliest phases of the nanoconstruct developmental process. Many in vitro models have therefore been set up by researchers with the aim of understanding the dynamic relationships between nanoconstructs and biological barriers; each model has its own pros and cons, and its experimental suitability is related to the needed information.

**Figure 1:**
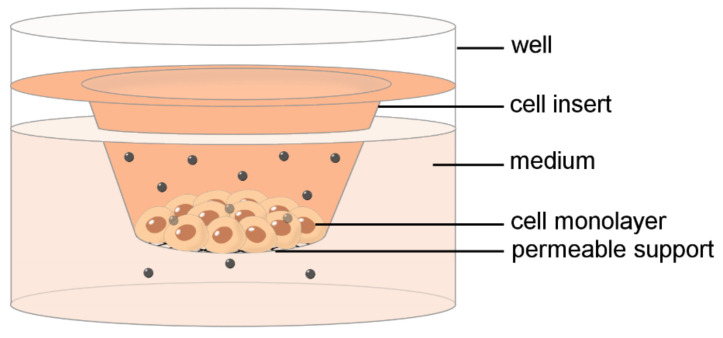
Schematic drawing of an in vitro 2D barrier model made of a culture well with a cell culture insert. Nanoparticles are administered in the medium in the cell insert and monitored for their passage to the lower chamber through the cell monolayer.

Conventional 2D cell cultures have been the mainstay of nanomedical research due to their simplicity, cost-effectiveness, and reproducibility. Cultured cells gave basic information on NP capability to cross the plasma membrane as well as the intracellular barriers, such as, e.g., the endosome membrane or the nuclear envelope; however, the information remains limited to single cells [11,12]. To better mimic the cytoarchitectural complexity of biological barriers, polarized cells have been grown as monolayers of either mono- or co-cultures on permeable supports (Figure 1), thus allowing the formation of cell–cell junctions and apical specialization and the assessment of the penetration capability of NPs across structured cellular barriers [13,14].

**Figure 2:**
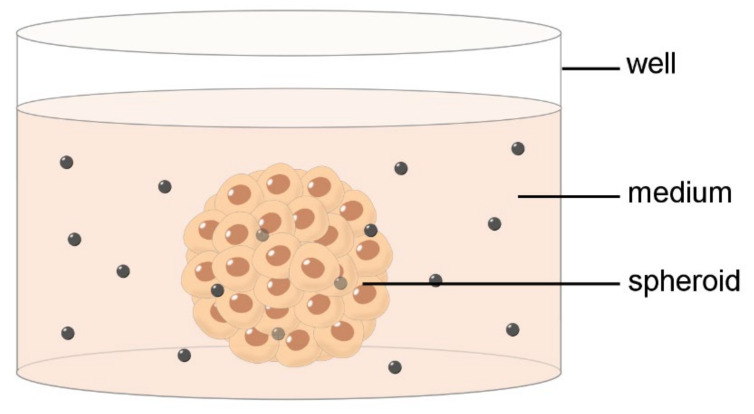
Schematic drawing of an in vitro 3D barrier model. Nanoparticles are administered in the medium and monitored for their penetration into the spheroid.

However, cell monolayers are also far from being realistic biological barriers as they lack both three-dimensionality and histological organization. Consequently, many 3D cell culture systems (Figure 2), often including the extracellular matrix, have been constructed [15,16]. Although 3D models are time-consuming, require trained handling, and has low reproducibility between research groups, they more closely mimic the normal cell–matrix and cell–cell interactions than 2D models, being thus more appropriate to test in vitro the NP delivery through complex biological barriers.

**Figure 3:**
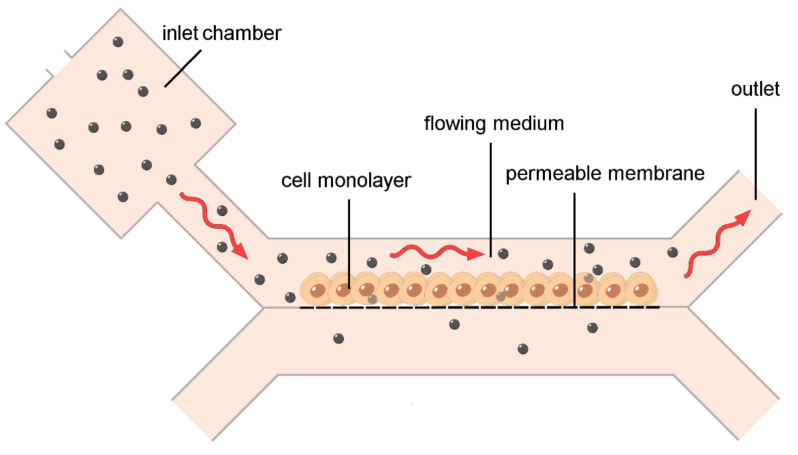
Schematic drawing of an in vitro barrier model under fluid dynamic conditions. Despite the high heterogeneity of devices described in the literature, the design is in principle the same for bioreactors and microfluidics. Nanoparticles are administered through the inlet chamber, move to the cells in the upper channel due to the flowing medium, and cross the cell monolayer toward the lower channel.

In vitro 2D and 3D modelling of biological barriers has received great advantage from the construction of bioreactors able to maintain cells and tissues under fluid dynamic conditions (Figure 3), thus mimicking the physiological flow occurring in the vasculature and extracellular milieu [17,18,19,20]. The microfluidic technologies enabled the construction of compartmentalized 3D cell culture devices connected by microchannels, where small volumes of fluids flow under strictly controlled conditions of chemical composition, temperature, and velocity [21] (Figure 3), thus providing nanomedical research with organs-on-chips suitable to reliably assess NP relocation through diverse biological barriers [22,23].

In this constantly evolving context, the aim of the present article is to provide an overview of the most representative and performing in vitro models of biological barriers set up for investigating the dynamic interaction of nanoconstructs for diagnostic or therapeutic applications. To the best of our knowledge, in the varied panorama of scientific literature, no review focusing on the utilization of in vitro barrier models in nanomedical research is available. Furthermore, with the aim of reducing in vivo experimentation, the present work will be helpful to nanotechnologists to identify the most suitable in vitro model for their experimental needs.

## 2. The Blood–Brain Barrier

The blood–brain barrier (BBB) is a selectively permeable system that regulates the flow of several molecules between the vascular blood stream and the extracellular fluid of the neural tissue in the central nervous system of most vertebrates. The BBB restricts the entry of more than 98% of small molecules and approximately 100% of large molecules [24], playing a key role in maintaining homeostasis and protecting the brain against pathogens and neurotoxins [25]. This extraordinarily selective barrier, also known as the neurovascular unit, is composed of endothelial cells, pericytes (laying in the endothelial cell basement membrane and encircling the microvasculature), and astrocytes whose end-feet enwrap the capillaries [26,27].

Because of its high selectivity, the BBB is the major obstacle for the development of therapeutic drugs for many brain diseases (e.g., cancer and neurodegenerative disorders). To address this issue, NPs represent a promising strategy to increase brain penetration and consequently drug delivery: due to their tunable properties, they can be easily modified to exploit the physiological transport mechanisms of the BBB [28].

Since the 1980s, in vitro models of the BBB have been developed and most of them are focused on mimicking the barrier in non-pathological brain conditions [29].

Many researchers have been using the in vitro system where an insert, consisting of microporous membrane filters, allows for simultaneously co-culturing different cell types, to test the uptake, trans- endothelial permeability, and effects of different type of NPs, according to their chemical-physical properties [30,31,32]. In the simplest configuration, endothelial cells are seeded on top of a micro-porous membrane and co-cultured with other neurovascular cells, commonly astrocytes, placed either on the bottom side of the membrane or directly onto the multiwell dishes [33,34,35,36,37]. More complex cell culture insert models may involve a triple co-culture where brain endothelial cells are cultured on top of the insert, and pericytes, astrocytes or mixed glial cells either on the bottom side or directly on the plate [38,39,40]. Cell insert co-culture systems have shown a great potential due to their ease of use, low cost, repeatability, and ability to reproduce the cross-talk between endothelial cells and neighboring elements. However, these models are oversimplified and associated with many challenges, such as the irregularity of the endothelial cell monolayer grown on a culture insert [41], the absence of the 3D environment of non-endothelial cells, and the excessive distance between the endothelial cells and astrocytes (10–50 μm vs. 40–80 nm in vivo spacing) that precludes close intercellular contacts and paracrine effects [42].

To overcome these restrictions, 3D co-culture models have been developed. Gromnicova and colleagues [43] used a 3D co-culture system in which brain endothelial cells (hCMEC/D3) were seeded on top of a 3D collagen gel containing human astrocytes to investigate the trans-endothelial delivery and astrocytes’ uptake of gold NPs coated with glucose and were able to exploit the glucose transporter located on the luminal surface of brain endothelial cells.

The multicellular BBB organoid is another reliable and predictive 3D in vitro platform useful to evaluate NPs’ uptake in the brain. In this model, endothelial cells, astrocytes, pericytes and other cell types are co-cultured in close contact with each other, forming cell–cell junctions and mimicking the integrity of the BBB [41]. As an example, Sokolova and colleagues [44] evaluated the penetration of ultra-small gold NPs across the BBB using a six-cell brain spheroid model composed of astrocytes, pericytes, endothelial cells, microglia, oligodendrocytes, and neurons.

Although these 2D and 3D static models are able to mimic some key properties of the BBB, they are still lacking the most relevant physiological conditions, i.e., the exposure of endothelial cells to shear stress by the blood flow. In fact, there is increasing evidence that the luminal flow present in brain capillaries may alter the interaction between NPs and endothelial cells and consequently the barrier penetration, not only by causing collision and rolling of NPs but even affecting endothelial cell phenotype, formation of intercellular tight junctions, and expression of specific membrane-bound receptors [45]. It is crucial to understand the correlation between fluid shear stress and NPs penetration because the physiological flow is often altered in pathological conditions, such as ischemic stroke and cancer.

In vitro dynamic systems have therefore been developed using the microfluidic technology, able to reproduce the physiological or pathophysiological brain microenvironment. A microfluidic-based BBB device commonly consists of a porous membrane (serving as a cell culture area) positioned in the middle of two channels aligned vertically or horizontally: two separate compartments are thus obtained that simulate the blood side and the brain side separated by an endothelial cell monolayer. These dynamic in vitro models have been widely used to investigate the ability of different kinds of native and functionalized NPs to interact and transit across the BBB [42,45,46,47]. For instance, Papademetriou and colleagues [45] utilized the microfluidic BBB model to evaluate the endothelial association and BBB translocation of liposomes conjugated with angiopep-2 (Ang-2), while Nowak and collaborators [48] studied the endothelial association and basolateral transport of carboxylated polystyrene NPs. An in vitro microvascular open model system using human brain endothelial cells offered researchers an innovative brain microvessel-on-a-chip suitable for measuring permeability to nanocarriers for therapeutic and diagnostic purposes [49].

Other researchers cultured more than one cell type into the microfluidic device in order to improve reliability of the in vitro BBB model, in an attempt to evaluate the capability of different NPs to penetrate the barrier. For instance, the flow-based in vitro BBB model constructed by Hudecz and collaborators [42] was a co-culture of brain capillary endothelial cells (hCMEC/D3) and primary human normal astrocytes.

A further in vitro BBB model involved the use of a cross linkable copolymer in order to coat and functionalize the microfluidic-based BBB chip channels with extracellular matrix proteins, thus enhancing BBB formation and allowing the translocation of transferrin-functionalized NPs [50]. Moreover, the BBB microvasculature can be also reproduced using a 3D network formed by the self-assembly of human endothelial cells obtained from pluripotent stem cells, brain pericytes, and astrocytes within a 3D fibrin hydrogel [51].

Although the majority of the in vitro BBB models were designed to reproduce the barrier in its integrity, some BBB models have also been developed to mimic a pathological condition characterized by a compromised barrier integrity. These models have been exploited to develop therapeutics tools against specific neurological disorders. For instance, Heggannavar and colleagues [37] provided evidence that paclitaxel-loaded poly-ε-caprolactone NPs conjugated with transferrin were able to easily cross the BBB model developed using human brain endothelial cells (HBMEC) cultured in the upper side of insert membrane and U87 glioma cells in the basolateral compartment of the insert. Tricinci and collaborators [52] developed a microfluidic device enabling a triple co-culture of human cerebral endothelial cells, astrocytes, and glioblastoma cells spheroids, demonstrating the suitability of superparamagnetic iron oxide NPs to cross the endothelial barrier and deliver dextran to tumor cells.

A summary of the in vitro BBB models cited in this chapter is reported in Table 1.

## 3. The Tumor Microenvironment Barrier

Tumor microenvironment (TME) is a complex and dynamic entity composed of the tumor parenchyma and a heterogeneous stromal compartment where immune cells, cancer-associated fibroblasts, blood and lymphatic vessels, and an extracellular matrix coexist and interact. This particular environment plays a key role in supporting the survival of cancer cells, by regulating their morphotype, signaling pathways, proliferation rate, and metastatic dissemination [53]. In principle, the hyper-permeable vasculature and the low lymphatic drainage of TME would be helpful for NP-mediated anticancer therapies; however, TME as a whole constitutes a hardly penetrable biological barrier due to several factors. The abnormal organization of the blood vessels and vessel compression due to proliferating cancer and stromal cells prevents a uniform NP distribution in the tumor mass; the high interstitial pressure and the great content in collagen fibers limit the extravasation and diffusion of NPs, causing their intravascular accumulation; hypoxia, acidosis and necrosis promote intrinsic drug resistance; and tumor-associated macrophages may internalize the NPs thus reducing their therapeutic effect [54]. Therefore, in vitro models closely mimicking the complex biological barrier of TME are essential to designing novel nanotechnology-based platforms for cancer treatment.

3D multicellular tumor models have been widely used in nanomedical research as a reliable in vitro TME barrier, especially because it has been demonstrated that 2D cell cultures may react to NPs in a manner far from that observed in a 3D structure [55,56,57].

Spheroids of prostate carcinoma cells or of breast cancer cells were used to test various formulations of liposomes [58] and gold NPs [59] for their penetration and distribution inside the tumor mass. HeLa cell spheroids allowed for evaluating the efficacy of quantum dots and doxorubicin-loaded synthetic micelles in comparison to conventional 2D cell culture, demonstrating their greater morphological and functional resemblance to in vivo tumor features, in terms of NP penetration and resistance to chemotherapeutics [60]. The efficacy in doxorubicin delivery was also studied in spheroids of human cervical carcinoma cells treated with triblock polymeric micelles [61] and in neuroblastoma cells spheroids treated with chitosan-poly(N-3-acrylamidophenylboronic acid) NPs [62], with positive results due to drug encapsulation and consistently with antitumor efficacy observed in vivo. Spheroids made of human prostate or kidney cancer cells were also used to test the efficacy of glycogen-small interfering RNA (siRNA) nanoconstructs in penetrating multicellular tumor mass and exerting gene silencing effect [63]. 3D tumor spheroids proved to be especially suitable to demonstrate the efficacy of ligand-mediated targeted nanocarriers able to release the encapsulated drugs in the tumor site following an external stimulus (e.g., heat, pH, or specific peptides) [64,65,66]. Sims and collaborators [67] used various spheroids made of different cancer cell types to estimate the penetration and distribution rate of poly(lactic-co-glycolic acid) (PLGA) NPs as a function of their surface-modification, hypo-vascularization of the tissue, and cancer cell type. A treatment with collagenase was used to experiment on human cervical carcinoma spheroids in order to investigate the modulating effects of the tight cell–cell interactions and extracellular matrix barrier in the penetration and distribution of polystyrene NPs [68].

A more complex spheroidal in vitro tumor model was set up by Ho and collaborators [69] by co-culturing glioblastoma cells and endothelial cells to mimic a tumor core coated by an endothelial layer to investigate the penetration potential of iron oxide NPs. Similarly, Priwitaningrum and collaborators [70] co-cultured breast or pancreas cancer cells with fibroblasts to mimic the intra-tumor stromal barrier; this model was used to investigate the penetration of differently sized and charged silica and PLGA NPs, which allowed for demonstrating how stromal cells act as a barrier against NP penetration.

As a further evolution of TME barrier modeling, matrix- and scaffold-based 3D in vitro tumor models have been developed in which cells adhere, proliferate, and spatially organize on naturally derived hydrogels or polymeric scaffolds, thus closely resembling a tumor mass. These complex models were used for the preclinical evaluation of various nanocarriers for anticancer drugs [71,72,73,74,75]. In order to mimic the delivery of extravasated NPs, Ng and Pun (2009) set up a perfusable 3D cell-matrix culture chamber made of cells cultured in Matrigel to compare the distribution of differently sized NPs introduced by pressure-induced flow (imitating the interstitial flow) or under static conditions, thus demonstrating the importance of a dynamic in vitro model [76].

Finally, with the advent of microfluidics, tumor-on-chip and TME-on-chip models have been developed, with microcircuits containing flowing fluids surrounded by spatially organized tumor cells, thus allowing the reproduction of complex cell–cell and cell–matrix interactions as well as the fluid dynamics occurring in vivo. These novel 3D systems were therefore used to analyze under flow conditions the biodistribution, delivery kinetics, and therapeutic efficacy of NPs designed to treat, e.g., melanoma [77], breast cancer [78,79,80,81], hepatocellular carcinoma [82], and colorectal cancer [83].

Interestingly, a TME-on-a-chip model allowing co-culture of tumor spheroids and macrophages in a 3D gel matrix was used to investigate the suitability of macrophages loaded with paclitaxel-encapsulated polymer NP as cellular drug-delivery agents able to deeply penetrate the tumor mass thanks to their own migrating capability [84].

Finally, a very simple in vitro model to assess nanocarrier penetration through the tumor tissue after extravasation has been recently described by McCormick and collaborators [85]: fluorescent NPs are monitored while moving into a tissue-mimetic microfluidic chip loaded with hydrogels (Matrigel and collagen I) and cell-sized microparticles.

A summary of the in vitro tumor microenvironment barrier models cited in this chapter is reported in Table 2.

## 4. The Endothelial Barrier

The endothelial barrier may be part of the BBB and TME barrier (as stated in the previous chapters) but not only. In fact, the extravasation allows systemically-administered NPs to leave the bloodstream and enter the neighboring tissue to play their diagnostic or therapeutic action. Quantitative studies on endothelial permeability to NPs are quite difficult in vivo but, during the last decade, the development of microfluidics has allowed for facing this crucial issue using in vitro models of the endothelial barrier.

Kim and collaborators [86] developed a microfluidic device consisting in a microporous polyester membrane sandwiched between two independent microfluidic channels; the cavities were lined with human umbilical vein endothelial cells and provided with electrodes to measure flow rate and endothelium shear stress. This in vitro model of endothelial barrier was used to investigate the translation rate of lipid-polymer NPs and the results compared with an in vivo model, thus demonstrating the reliability of the system.

An in vitro biomimetic microfluidic blood vessel model lined with a fenestrated monolayer of human umbilical vein endothelial cells, mimicking the tumor endothelial monolayer barrier, and surrounded by a fibrin hydrogel modeling the extracellular space was set up to test the suitability of dextrin-conjugated graphene oxide nanocarriers for anti-cancer drug delivery [87].

A microfluidic device lined with a monolayer of human umbilical vein endothelial cells was used to tune vascular permeability with angiopoietin 1 and cyclic adenosine monophosphate treatments, thus simulating healthy and tumor endothelium and allowing for quantifying the diffusional permeability of the endothelial barrier to differently sized polystyrene NPs [88]. To study NP extravasation from the leaky tumor vasculature and accumulation in tumor tissues, Wang and collaborators [89] set up a microfluidic tumor-vasculature-on-a-chip consisting of channels lined with human endothelial cells made permeable with tumor necrosis factor and surrounded by 3D spheroids immersed in a dense extracellular matrix.

Using a microfluidic device, Moore and collaborators [90] demonstrated that NPs may also cross the capillary wall due to the monocyte/macrophage cells adhering to the endothelial monolayer: under physiological flow, these cells act as shuttles by internalizing the NPs and delivering them across the endothelium.

The role of blood flow in targeting specific endothelial markers by nanoconstructs intended for imaging or therapeutic purposes was investigated by analyzing the binding affinity of polystyrene NPs with different shapes and coating under dynamic flow conditions in microfluidic channels lined with human endothelial cells [91].

An innovative microfluidic device enabling the build of a 3D vascular network consisting of human umbilical vein endothelial cells and lung fibroblasts on a fibrin scaffold was used to demonstrate the suitability of liposomes functionalized with anti-ICAM-1 antibody to target inflamed endothelium [92].

Finally, an original functional improvement for microfluidic models of endothelial barriers consisting of microchannels bordered by a collagen matrix was proposed by Schuerle and collaborators [93]: they enhanced local fluid convection using bacteria-inspired microrobots or swarms of magnetotactic bacteria as propellers, thus obtaining an increased NP extravasation and matrix penetration.

A summary of the in vitro endothelial barrier models cited in this chapter is reported in Table 3.

## 5. The Lung Barrier

The lung is the respiratory organ of terrestrial vertebrates, and it is characterized by a large surface area devoted to the transport/modification of air (bronchial tree) and gas exchange (alveoli). The bronchial surface is lined by the respiratory epithelium, mainly made of a ciliated pseudostratified columnar epithelium with mucin-producing goblet cells (both cell types taking part in the mucociliary clearance process), and it lies on a richly vascularized connective layer. Gas exchange takes place in the alveoli through the air–blood barrier, which is formed by the alveolar epithelium, the capillary endothelium, and the extracellular matrix in between.

Due to the continuous airflow and the close proximity to the capillary bed, the respiratory and alveolar epithelia are important biological barriers not only for their physiological functions but also for environmental health assessments and drug delivery. The lung barrier has therefore been widely explored for the therapeutic application of nanocarriers via inhalation or tracheal instillation. The capability of nanoconstructs to cross this barrier is therefore basic information and several in vitro models have been set up to this aim.

The first attempts to mimic the lung barrier in vitro to investigate the capability of NPs to pass across the respiratory epithelium implied the use of simple 2D cell cultures. Geys and collaborators [94] used a human alveolar epithelial cell line, a human bronchial epithelial cell line and rat primary type II pneumocytes to form intact monolayers onto cell culture inserts, and analyzed the translocation of fluorescent polystyrene NPs through the cell monolayer. Similarly, George and collaborators [95] compared the suitability of different human bronchial and alveolar epithelial cell monolayers to study the crossing capability of fluorescently labelled silica NPs.

However, this simple in vitro model is very far for the complexity of the lung epithelia. To improve the resemblance to epithelial airway barrier, a triple co-culture cell model consisting of epithelial, macrophagic, and dendritic cells was established and utilized to investigate the location of gold NPs [96]. The use of a 3D human bronchial epithelial model with a mucociliary apparatus cultured at the air–liquid interface allowed for an understanding of the key role played by the mucus and cilia to prevent cerium oxide NPs from reaching the epithelial cells and crossing the barrier [97]. As further proof of the importance of the mucociliary apparatus in modelling a reliable lung epithelial barrier, it is worth recalling that the same cerium oxide NPs were internalized in a monolayer of A549 lung cells cultured at the air–liquid interface [98]. Interestingly, in vitro model demonstrated that mucins prevent from crossing epithelial barriers not only NPs but also drugs [99].

A significant advancement in the suitability of in vitro respiratory barrier models came from the development of a biomimetic dynamic microsystem constituted by a co-culture of epithelial and endothelial cells located on either side of a porous polymeric membrane, thus mimicking the alveolar–capillary interface [100]. The organ-on-a-chip technology opened the way to in vitro models more and more complex and similar to in vivo structures, which were used to investigate the capability of nanoparticulates to interact with the lung epithelial barrier. Of particular interest is the 3D human lung-on-a-chip model, where a Matrigel layer (simulating the extracellular matrix) separates the two cell monolayers [101]. A recent study focused on the importance of dynamic conditions mimicking breathing in the functionality of in vitro lung barriers. In fact, Doryab and collaborators [102] set up a lung bioreactor where alveolar cells were grown at the air–liquid interface adhering on a biomimetic co-polymeric membrane, under periodic stretching. By this dynamic system, the authors demonstrated that the cellular uptake of NPs is significantly increased by the physiological stretching and alerted us to a possible underestimation of the transbarrier transport of nanoparticulates in static models.

A summary of the in vitro lung barrier models cited in this chapter is reported in Table 4.

## 6. The Intestinal Barrier

The intestinal barrier is devoted to absorbing nutrients and, at the same time, to protecting the organism from potentially noxious exogenous factors (e.g., pathogens, xenobiotics). The intestinal barrier is quite complex, being made of columnar epithelial cells (enterocytes), mucus-secreting goblets cells, and various types of endocrine and immune cells; the luminal surface is covered by a mucus layer colonized by the microbiota [103]. The high, although selective, permeability of the intestinal barrier and its relatively easy accessibility made it very interesting as a suitable administration route for many therapeutic agents for both local and systemic targeting, due to the immediate passage of the absorbed agents into the bloodstream.

In nanomedicine, several studies focused on nanocarriers as tools for oral delivery of nanoencapsulated therapeutic peptides and vaccines. To this aim, in vivo approaches have been often flanked by the use of in vitro models to investigate the nanocarrier translocation through the intestinal barrier. Although most of the internalization tests have been performed in conventional 2D cultures, some authors improved the in vitro systems with the aim of better mimicking the in vivo barrier complexity.

The influence of mucus in NP transport through the intestinal mucosa was highlighted in a study by Jin and collaborators [104]. In fact, an enterocyte/goblet co-cultured cell monolayer was set up to better simulate the intestinal epithelium, and it showed an enhanced transport ability of chitosan NPs for oral delivery of insulin in comparison to a conventional enterocyte cell monolayer. A microfluidic in vitro model of the gastrointestinal mucus barrier demonstrated that the interaction between mucins and cationic chitosan/siRNA nanocarriers resulted in their disassembly and consequent siRNA diffusion across the mucin barrier [105]. More recently, a mucus-on-chip microfluidic device was fabricated by using two parallel microchannels, one filled with mucin and the other one with flowing NP solution, providing detailed information on the dynamic interaction of polystyrene NPs with mucus, which resulted especially related to the nanoconstruct surface properties [106].

Des Rieux and collaborators [107] used a co-culture of Caco-2 cells and human RajiB lymphoblast-like on culture inserts (as an in vitro model of human follicle-associated epithelium) to investigate the oral delivery of nanoencapsulated drugs, demonstrating the positive role of Microfold cells (M cells) in the barrier crossing. The same authors optimized this in vitro model by inverting the culture orientation to investigate the transcellular transport of polystyrene NPs [108]. Similarly, Kadiyala and collaborators [109] demonstrated that the transport of DNA-chitosan NPs through the M-cell co-culture model is much more efficient than in conventional intestinal epithelial monolayers, supporting the importance of the presence of such cellular component for the reliability of in vitro intestinal barrier models.

According to the above findings, a triple co-culture system constituted by Caco-2 cells, mucus-secreting goblet cells, and M cells demonstrated that the permeability to polystyrene NPs of this in vitro intestinal barrier was strongly increased by the presence of mucus and that crossing occurred preferentially via specialized M cells [110]. Importantly, these results were consistent with in vivo and ex vivo tests. The same triple co-culture system was successfully used to assess the suitability of PLGA-based NPs for the oral delivery of insulin [111].

Different in vitro models of intestinal barrier (i.e., a monocultures of Caco-2 cells, a co-culture of Caco-2 cells with mucus-secreting cells, and a co-culture of Caco-2 with RajiB cells) were used by to evaluate the translocation of titanium dioxide NPs [112]. These NPs proved to cross the in vitro barrier only in the co-culture with RajiB cells, further demonstrating the key role of the follicle-associated epithelium overlying Peyer’s patches in NP uptake [113].

Different intestinal barrier models—from the simple Caco-2 cell monolayer to the co-culture made of Caco-2 and mucus-secreting cells to a tri-culture made of Caco-2, mucus-secreting and M cells—were also used to assess the crossing capability of polystyrene NPs characterized by various sizes and surface charges [114]. The amount of translocated NPs was significantly affected by the barrier model type, underlying the role of the different cellular components in determining barrier permeability. In addition, the enterocyte/mucus secreting in vitro system revealed that the permeability of the intestinal barrier also depends on the surface chemical feature of the NPs, suggesting that the effect of gastric digestion on the intestinal translocation should be considered when testing NPs intended for oral administration [115].

In recent years, many efforts have been made to manufacture intestine-on-a-chip in order to construct an intestinal model reliably simulating the in vivo barrier [116]. An interesting example is the new membrane-based microfluidic platform mimicking the intestine wall set up by Mitxelena-Iribarren and collaborators [117] to assess the suitability of lipid nanocarriers designed for oral delivery of anti-cancer drugs. However, these models have been scarcely used in nanomedicine.

A summary of the in vitro intestinal barrier models cited in this chapter is reported in Table 5.

## 7. The Skin Barrier

The skin is one of the most effective biological defense barriers of the organism, protecting the entire body surface from chemical, physical and mechanical insults as well as from the loss of water. It is composed of two layers: the epidermis (i.e., the most superficial layer of stratified keratinized squamous epithelium) and the underlying dermis (a highly vascularized and permeable layer of connective tissue). At the same time, the skin is a major way of communication of the organism with its environment; consequently, it is an interesting route to administer drugs for both local and systemic treatments. Many nanocarriers have been formulated for transdermal delivery and diverse in vitro skin barrier models have been made-up to mimic the complex skin cytoarchitecture to test the suitability of novel nanoconstructs.

Commercially available 3D skin models, such as Episkin, EpiDerm™, and Neoderm^®^-ED, made of in vitro reconstructed human epidermis from normal human keratinocytes, were frequently used to test the transdermal penetration capability of different NPs [118,119,120,121].

A simplified 3D skin model was manufactured by printing a blank collagen layer and 3T3 fibroblasts alternately in a layer-by-layer fashion, to verify the transdermal penetration ability of silica NPs [122]. Similarly, the repair and regeneration capability of zinc sulfide NPs was assessed on a 3D skin model made of a hydrogel scaffold seeded with rat fibroblasts, and validated by in vivo wound healing tests on rat skin [123]. In addition, the potential of sprayable silver NPs as an antibacterial cutaneous barrier was tested on a skin model made of human skin fibroblasts embedded within collagen hydrogel [124]. However, these models should be considered as dermis models instead of skin models.

A more complex skin model, constituted of fibroblasts embedded in type I collagen and human keratinocytes seeded on the collagen matrix surface, was used to investigate the dynamics of drug delivery by core-multishell nanoconstructs [125].

The antibacterial activity of silver-modified SiO2-CaO mesoporous bioactive glass NPs was assessed on an original 3D tissue-engineered model of infected skin where a human decellularized dermis (as a base scaffold) was seeded with human dermal fibroblasts and keratinocytes, and subsequently infected with *Pseudomonas aeruginosa* or *Staphylococcus aureus* [126]. A similar 3D skin model was used to assess the antibacterial and pro-angiogenic properties of copper-containing mesoporous glass NPs with the aim of curing chronic wounds [127].

More recently, an innovative 3D bacterial biofilm/human keratinocyte clusteroid co-culture platform was used as an in vitro model of microbial biofilm on human skin to assess the antibacterial efficiency of ciprofloxacin-loaded Carbopol nanogel particles on clearing *S. aureus* and *P. aeruginosa* [128].

To evaluate the therapeutic efficacy of nanocarriers in delivering anticancer drugs, a full-thickness bioengineered skin containing melanoma SK-MEL-19 cells was fabricated as a 3D skin cancer model. In detail, the dermis was produced with fibroblasts embedded in a type I collagen matrix, while the epidermis was grown above this layer using keratinocytes, adding melanoma cells to mimic cancer [129,130].

A summary of the in vitro skin barrier models cited in this chapter is reported in Table 6.

## 8. Conclusions

Literature data demonstrate how many efforts have been made by researchers to develop in vitro models of biological barriers to study, under strictly controlled conditions, the translocation of nanoconstructs intended for medical purposes. Some biological barriers received more attention than others due to a greater nanomedical interest or simply because human ex vivo models are easily available, as in the case of the skin. It is worth noting that, in addition to those mentioned in the present review, other in vitro models have been set up to mimic, e.g., the placental [131,132,133], renal [134,135,136], or ocular barrier [137,138,139,140]; however, so far, they were not applied in the nanotechnological field or their use was limited to nanotoxicological studies. It is easy to foresee that they will also be used in nanomedical studies in the near future.

We believe that the present review article will help scientists to approach the barrier model world, and to select the most appropriate systems for their research. The in vitro barrier models often achieved good structural and functional similarity to the in vivo barriers, and the greater the complexity of the model in terms of cell types and flow dynamics, the more reliable were the results obtained. In this view, fluid dynamic devices and especially microfluidics did represent a crucial step forward. However, apart from rare simplified microfluidic devices [141], manufacturing microfluidic cell culture systems is difficult and restricted to a small number of research groups, which so far limited their wide application in nanomedical experimentation.

Thanks to the continuous technological advancement and their economical and ethical benefits, the in vitro barrier models will become more and more attractive to researchers in nanomedicine in the years to come. Reducing the cost and fabrication complexity of dynamic culture systems will be crucial to boost nanomedical research because simple, reproducible, and functionally and anatomically realistic in vitro barrier models will offer a valuable tool for NP development and facilitate preclinical studies on NP biodistribution and pharmacokinetics.

## Figures and Tables

**Table 1:** Summary of in vitro BBB models used for nanomedical studies.

BBB MODELS			
Model	Cell types	Nanoparticles	References
Cell culture insert	Co-culture of bovine-brain endothelial cells and rat astrocytes	Gold NPs; Gelatin-siloxane NPs; Polymer NPs	[30,34,35]
Cell culture insert	Co-culture of HBMEC human brain-microvascular endothelial cells and human astrocytes	Polymer NPs; Lipid NPs	[31,32]
Cell culture insert	Co-culture of rat brain capillary endothelial cells and rat astrocytes	Cationic bovine serum albumin NPs	[33]
Cell culture insert	Co-culture of RBE4 rat brain endothelial cells and C6 rat astrocytoma cells	PEG-PLGA NPs	[36]
Cell culture insert	Co-culture of HBMEC human brain microvascular endothelial cells and U87 MG human glioblastoma cells	Poly-ε-caprolactone NPs	[37]
Cell culture insert	Co-culture of rat brain endothelial cells and rat brain pericytes	Silica NPs	[39]
Cell culture insert	Co-culture of rat brain endothelial cells, rat brain pericytes and rat glial cells	Niosomes; Silver NPs	[38,40]
Spheroid	Co-culture of human astrocytes, pericytes, endothelial cells, microglia cells, oligodendrocytes and neurons	Gold NPs	[44]
Spheroid	Co-culture of hCMEC/D3 human brain endothelial cells, astrocytes, and U87 MG human gliobastoma cells	Superparamagnetic iron oxide NPs	[52]
Microfluidic device	bEnd.3 mouse brain endothelial cells	Liposomes; Polystyrene NPs	[45,47]
Microfluidic device	hCMEC/D3 human brain endothelial cells	Polymer NPs; Polystyrene NPs	[46,48]
Microfluidic device	Co-culture of hCMEC/D3 human brain endothelial cells and human astrocytes	Polystyrene NPs	[42]
Microfluidic device	Co-culture of hCMEC/D3 human brain endothelial cells, perycites and astrocytes	Silicon NPs	[50]
Microfluidic device	Co-culture of human-induced pluripotent stem cell-derived endothelial cells, primary brain pericytes and astrocytes	Polymer NPs	[51]

**Table 2:** Summary of in vitro tumor microenvironment barrier models used for nanomedical studies.

TUMOR MICROENVIRONMENT BARRIER			
Model	Cell types	Nanoparticles	References
Spheroid	LNCap-LN3 human prostate cancer cells	Liposomes	[58]
Spheroid	MCF-7 human breast cancer cells	Gold NPs	[58,59]
Spheroid	HeLa human cervical cancer cells	Quantum dots; Synthetic micelles; Gold core-mesoporous silica shell rod-like NPs encapsulated in PLGA microparticles; PLGA NPs	[60,66,67]
Spheroid	SiHa human cervical cancer cells	Triblock copolymers micelles; Polystyrene NPs; PLGA NPs	[61,67,68]
Spheroid	SH-SY5Y human neuroblastoma cells	Chitosan NPs	[62]
Spheroid	293T-luc human kidney epithelial cells; PC3 human prostate epithelial cancer cells	Glycogen-ethylenediamine NPs	[63]
Spheroid	HCT-116 human colorectal carcinoma; Human dermal fibroblasts	Polymeric micelles	[64]
Spheroid	U87-MG human glioma cells; Primary human dermal fibroblasts	PLGA-PEG NPs	[65]
Spheroid	Co-culture of RG2 rat glioblastoma cells and bovine-pulmonary arterial endothelial cells	Iron oxide NPs	[69]
Spheroid	4T1 mouse breast cancer cells and 3T3 murine fibroblasts; co-culture of Panc-1 human pancreatic cancer cells and human primary pancreatic stellate cells; Co-culture of MDA-MB-231 human breast tumor cells and BJ-hTert human fibroblasts	Silica NPs	[70]
3D matrix-based cell culture	HeLa human cervical cancer cells	PLGA-PEG NPs	[73]
3D matrix-based cell culture	LNCaP human prostate cancer cells	Polymer NPs	[71]
3D matrix-based cell culture	HT1080 human fibrosarcoma cells; Primary human dermal fibroblasts	Polystyrene NPs	[72]
3D matrix-based cell culture	95-D human lung cancer cells; HCT116 human colon cancer cells; U87 human glioblastoma cells	Polymicelles	[74]
3D matrix-based cell culture	Co-culture of normal human mammary fibroblasts and MCF10 human epithelial breast cells; Co-culture of cancer associated fibroblasts and MCF7 human breast adenocarcinoma cells	PLGA-PEG NPs	[75]
3D matrix-based cell culture	3T3 mouse fibroblasts; MDCK dog kidney cells	Carboxylic acid-based NPs	[76]
Microfluidic device	MDA-MB-435 human melanoma cells	Gold NPs	[77]
Microfluidic device	Co-culture of MCF-7 human breast cancer cells and human microvascular endothelial cells	Gold NPs	[78]
Microfluidic device	Co-culture of MCF-7 human breast cancer cells and human primary adipose-derived stromal cells	Gold NPs	[79]
Microfluidic device	Co-culture of primary human breast tumor associated endothelial cells and MCF-7 or MDA-MB-231 human breast cancer cells	Liposomes	[80]
Microfluidic device	Co-culture of HUVEC primary human umbilical vein endothelial cells and T47D or BT549 human breast cancer cells	Carbon dots	[81]
Microfluidic device	HepG2 human hepatocellular carcinoma cells	Polystyrene NPs	[82]
Microfluidic device	Co-culture of HCT-116 human colorectal carcinoma and human colonic microvascular endothelial cells	Dendrimer NPs	[83]
Microfluidic device	Co-culture of SKOV3 human ovarian adenocarcinoma cells and RAW 264.7 murine macrophage cells	Polymer NPs	[84]
Microfluidic device	Cell-mimetic microparticles	Polystyrene NPs	[85]

**Table 3:** Summary of in vitro endothelial barrier models used for nanomedical studies.

ENDOTHELIAL BARRIER			
Model	Cell types	Nanoparticles	References
Microfluidic device	HUVEC human umbilical vein endothelial cells	Gold nanocrystals; Lipid–PLGA NPs; Graphene-oxide NPs; Polystyrene NPs	[86,87,88]
Microfluidic device	Co-culture of HUVEC human umbilical vein endothelial cells and SKOV3 human ovarian cancer cells	Liposomes; PLGA NPs	[89]
Microfluidic device	Co-culture of J774A.1 mouse monocytes/macrophages and primary mouse lung endothelial cells	Silica NPs	[90]
Microfluidic device	hCMEC/D3 human cerebral microvascular endothelial cell	Polystyrene NPs	[91]
Microfluidic device	Co-culture of HUVEC human umbilical vein endothelial cells and primary normal human lung fibroblasts; Co-culture of hASC human adipose-derived stem cells and hAMEC primary human adipose microvascular endothelial cells; Co-culture of primary human retinal endothelial cells, primary human ocular choroid fibroblasts and induced pluripotent stem cell-derived human retinal pigment epithelial cells; Co-culture of HUVEC human umbilical vein endothelial cells, primary normal human lung fibroblasts and A549 human lung adenocarcinoma cells	Liposomes	[92]
Microfluidic device	Bacteria-like microrobots	Carboxylate-modified NPs	[93]

**Table 4:** Summary of in vitro lung barrier models used for nanomedical studies.

LUNG BARRIER			
Model	Cell types	Nanoparticles	References
Cell culture insert	A549 human alveolar epithelial cells; Calu-3 human bronchial epithelial cells; NCI-H292 human bronchial epithelial cells; Primary rat type II pneumocytes	Polystyrene NPs; Cerium oxide NPs; Silica NPs	[94,95,98]
Cell culture insert	Co-culture of A549 human alveolar epithelial cells, human blood monocyte derived macrophages and human dendritic cells	Gold NPs	[96]
Cell culture insert	Fully differentiated bronchial epithelial MucilAir™ model	Cerium oxide NPs	[97]
Microfluidic device	Co-culture of A549 human alveolar epithelial cells and E10 murine pulmonary microvascular endothelial cells	Silica NPs; Quantum dots; Iron NPs; Polystyrene NPs; Carbon nanotubes; Gold NPs	[100]
Microfluidic device	Co-culture of HUVEC human umbilical vein endothelial cells and immortalized human alveolar epithelial cells	Titanium oxide NPs; Zinc oxide NPs	[101]
Bioreactor	A549 human alveolar epithelial cells; 16HBE14o−human bronchial epithelial cells	Polystyrene NPs	[102]

**Table 5:** Summary of in vitro intestinal barrier models used for nanomedical studies.

INTESTINAL BARRIER			
Model	Cell types	Nanoparticles	References
Cell culture insert	Co-culture of Caco-2 human colorectal adenocarcinoma cells and HT29-MTX human colon goblet cells	Chitosan NPs; Polystyrene NPs	[104,114,115]
Cell culture insert	Co-culture of Caco-2 human colorectal adenocarcinoma cells and Raji B human Burkitt’s lymphoma cells	Latex NPs; Polystyrene NPs	[107,108,113]
Cell culture insert	Co-culture of Caco-2 human colorectal adenocarcinoma cells and mouse isolated lymphocytes from Peyer’s patches	Chitosan NPs	[109]
Cell culture insert	Co-culture of Caco-2 human colorectal adenocarcinoma cells, HT29-MTX human colon goblet cells and Raji B human Burkitt’s lymphoma cells	Polystyrene NPs; PLGA NPs	[110,111,114,115]
Cell culture insert	Caco-2 human colorectal adenocarcinoma cells; Co-culture of Caco-2 and HT29-MTX human colon goblet cells; Co-culture of Caco-2 and Raji B human Burkitt’s lymphoma cells	Titanium oxide NPs; Polystyrene NPs	[112,114,115]
Microfluidic device	Porcine mucins	Chitosan NPs; Polystyrene NPs	[105,106]
Microfluidic device	Co-culture of Caco-2 human colorectal adenocarcinoma cells and U-2 OS human osteosarcoma cells	Lecithin-based NPs	[117]

**Table 6:** Summary of in vitro skin barrier models used for nanomedical studies.

SKIN BARRIER			
Model	Cell types	Nanoparticles	References
3D model	Reconstructed human epidermis from normal keratinocytes	Solid-lipid NPs; Gold NPs; Copper- and zinc-based NP; Lipid NPs	[118,119,120,121]
3D model	3T3 murine fibroblasts	Silica NPs	[122]
3D model	Primary rat skin fibroblasts	Zinc-based NPs	[123]
3D model	Primary human skin fibroblasts	Silver NPs	[124]
3D model	Co-culture of primary human keratinocytes and primary human dermal fibroblasts	Core-multishell NPs	[125]
3D model	Co-culture of primary human dermal fibroblasts and HaCaT human keratinocytes	Glass NPs	[126,127]
3D model	Co-culture of HaCaT human keratinocytes, *S. aureus* and *P. aeruginosa*	Carbopol nanogel particles	[128]
3D model	Co-culture of primary human normal fibroblasts, primary human normal keratinocytes and SK-MEL-19 human melanoma cells	Tributyrin-containing NPs; Nanoemulsions	[129,130]
